# Aging Might Increase the Incidence of Infection from Permanent Pacemaker Implantation

**DOI:** 10.1155/2013/943416

**Published:** 2013-11-28

**Authors:** Yun Lin, Zhi Zhong Li, Jingmei Zhang, Jinrong Zhang, Qian Fan, Jie Du

**Affiliations:** ^1^Department of Cardiology, Beijing Anzhen Hospital, Capital Medical University, Beijing 100029, China; ^2^The Key Laboratory of Remodeling-Related Cardiovascular Diseases, Capital Medical University, Ministry of Education, Beijing 100029, China; ^3^Beijing Institute of Heart, Lung and Blood Vessel Disease, Beijing 100029, China

## Abstract

*Aim*. The elderly are the major population receiving the implantation of a permanent pacemaker (PPM). Infection is a devastating complication. The present study is to verify the relationship between age and PPM implantation infection. *Methods*. All patients (162 adult and 292 elder patients) received the implantation of PPM. Subcutaneous tissue samples solution was collected in three time points, the first sample was got at skin incision, and the second sample was got when the PPM had been implanted. And the third sample was got after 0.9% NaCl quick rinse. And the tissue solutions were cultured. If culture results are positive, it is considered as evidence of the presence of bacteria in pocket in operation of PPM implantation. *Results*. The data demonstrated that compared with that in the adult patients, subcutaneous bacterial survival rate was higher significantly in the elderly. *Staphylococcus epidermidis *is the major bacterial strain. The rinse decreased subcutaneous bacterial survival rates in the adult group. *Conclusion*. With the age increasing, PPM implantation might be easier to result in infection. Simple rinse can prevent implantation infection significantly. However, age alleviated the protective effects of rinse. Therefore, we should pay more attention to post implantation infection in the elderly.

## 1. Introduction

Implantation of a permanent pacemaker (PPM) has been widely accepted and implemented worldwide as the treatment of choice for bradyarrhythmias [1]. Infection in a PPM is a serious complication. It may occur either as a surgical site infection (SSI), occurring within 1 year after implantation, or as late-onset lead endocarditis. Pacemaker implantation rates are on the rise worldwide, and the population of patients living with a PM is growing [2].

Infection is a devastating complication of PPM/ICD use. Rates of infection after system placement have varied considerably, from 0.13% to 19.9%, and antimicrobial therapy alone (without removal of the entire system) is complicated by mortality and frequent infection relapse. Thus, the prevailing opinion is that the optimal management of PPM/ICD infection includes complete removal of the device and leads coupled with antimicrobial therapy. However, this treatment will result in serious hurt to patients [2, 3]. Therefore, the best treatment is to prevent infection instead of surgery and antimicrobial therapy after infection.

The elderly are the major population of PPM implantation [4]. The previous data demonstrated that aging exerted the vital effects on cardiovascular diseases [5, 6]. Then is aging an independent risk factor to PPM implantation? Does aging retard or promote infection after PPM implantation? As to the question, the results from prior studies were contradictory. Some studies indicated that infection rate increased with aging, or there is not a relationship between age and infection rate. Surprisingly, several researches got the inverse results, which demonstrated that infection tend's to occur in the adult [7].

Obviously, most PPM's were implanted in the elderly [4, 7]. Additionally, implantation-induced infection is the most serious complication of this kind of treatment [8, 9].

According to the above confusion, our study was designed to test the following points: (1) the relationship between age and the incidence of infection, (2) the technique to decrease the occurrence of PPM-induced infection, and (3) the effects of age on infection-depressed treatment. It had been well known that implantation infection depends on the bacteria number surviving in the implantation site. Therefore, in the present study, the tissue samples were got from the implantation site, and samples were cultured in vitro. The culture results were used as the markers to evaluate the possibility of infection occurrence.

## 2. Methods

The clinical trial was carried out in accordance with The Declaration of Helsinki (DoH) of the WMA [10]. The study protocol was approved by the Institutional Ethics Committee of the Beijing Anzhen Hospital-Affiliate of Capital Medical University. After full disclosure of the study's purpose, nature, and inherent risks of participation, all subjects gave written informed consent prior to enrollment.

### 2.1. Inclusion and Exclusion Criteria of PPM Implantation Patients

**Figure 1:**
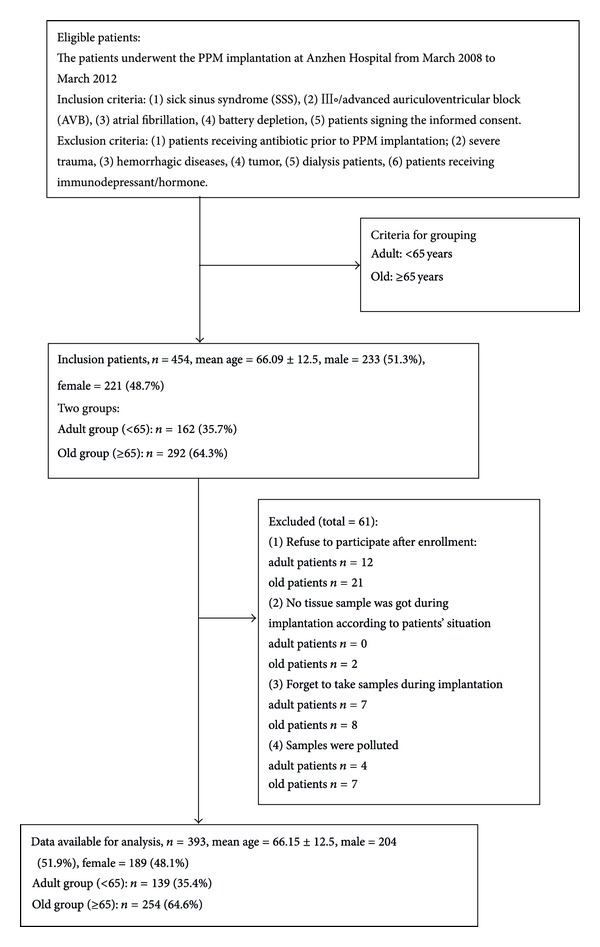


All patients who underwent PM (single or dual-chamber) implantation or reoperation with changes in hardware between March 2008 and March 2012 in Beijing Anzhen Hospital were included in the study. As shown in Figure 1, 454 patients were enrolled in the study. Inclusion criteria included (1) sick sinus syndrome (SSS), mean heart rate ⩽40 with significant clinical symptom; (2) third or second degree type 2 auriculoventricular block; (3) atrial fibrillation with 3 s RR interval and significant clinical symptom, or 5 s RR interval; (4) battery depletion; and (5) patients who signed the informed consent.

Exclusion criteria included (1) patients received antibiotic prior to PPM implantation within 72 h prior to implantation, (2) severe trauma, (3) hemorrhagic diseases, (4) tumor, (5) patients who received dialysis treatment, and (6) patients who received immunodepressant/hormone. According to the above criteria, as shown in Figure 1, 454 patients were enrolled in the study.

### 2.2. Data Collection Implantation Procedure

Demographic and clinical data were collected from the Beijing Anzhen Hospital medical records. The demographic data consisted of patient age, gender, residence, and payment types. Clinical variables of interest included evidence of presence of high blood pressure (HBP), diabetes, and coronary artery disease. In addition, patient diagnosis, PPM device type, number of implantation were recorded in the study.

The method of implantation was published elsewhere. In addition, antisepsis was performed immediately before surgery; the skin was painted with two solutions, successively: aqueous povidone iodine 10% solution followed by a povidone iodine 7.5% solution. Implantations were performed by the same operators (Yun Lin) under local anaesthesia and conscious sedation. Patients all received the same antibiotic prophylaxis. Clopidogrel therapy was systematically withdrawn 6 days before the implantation and treatment with vitamin K antagonists and heparin was discontinued at least 3 days and 6 h, respectively, before the implantation procedure. All patients had an international normalized ratio (INR) on the day of surgery.

All devices were implanted subcutaneously. Both procedure (skin to skin) and fluoroscopy time were systematically measured by a registered nurse.

### 2.3. Tissue Samples Collection and Bacterial Culture

Subcutaneous tissue samples solution was collected in three time points, the first sample was got at skin incision, and the second sample was got when the PPM had been implanted. And the third sample was got after saline rinse (0.9% NaCl quick rinse). With a sterile cotton swab dipped in a deep pocket of tissue fluid, cotton tip swab to ensure more than 75% area is infiltration. Within 30 minutes, samples were inoculated in broth. After enrichment, culture was inoculated in sheep blood agar and MacConkey agar to cultivate bacteria growth. The results were then used to determine the presence of bacteria, and for bacterial identification and drug susceptibility testing.

### 2.4. Saline Rinse and Tissue Solutions Bacterial Culture

In the previous study, we found that there were not significant differences among rinses of iodophors, antibiotic, and saline (30 mL within 2 s). However, the study also indicated, the rinses decreased the positive ratio of bacterial culture. In the present study, we collected the tissue solutions in the deep pockets (the third time point) and compared the positive ratio of bacterial culture between adult and elderly group.

### 2.5. Statistical Analysis

All values are presented as means ± SEM or ratio. Data were subjected to chi-square analysis and post-hoc Student's *t*, tests. All statistics were calculated utilizing Graphpad Prism 5.0. *P* values <0.05 were considered statistically significant.

## 3. Results

162 adult and 292 elderly PPM implantation patients were enrolled in the clinical trial. Of 454 patients, 12 adult and 21 elderly patients refused participation after enrollment, and 7 elderly patients discontinued participation due to severe situation during operation. Operators forgot to get the tissue solution from 9 adult and 8 old patients. In addition, the sample solutions from 10 adult and 10 old patients were polluted. As shown in Figure 1, data available were got from 131 adult and 262 old patients.  Table 1 lists all demographic data, baseline statistics, cardiovascular risk profile, and implanted PPM characters. The major differences between the adult and elderly patient populations involved the incidence of high blood pressure, diabetes, and coronary artery disease.

### 3.1. Age Increased the Positive Ratio of Bacterial Culture

In the previous study, there is a significant positive relationship between the positive ratio of tissue solution bacterial culture and PPM infection. As shown in Table 2, gender, blood pressure, and coronary artery disease did not increase positive ratio of bacterial culture significantly. Diabetes mellitus increased the positive ratio of bacterial culture at the first time point, which had been reported in the previous researches. Interestingly, in the present study, aging resulted in the increasing of positive ratio of bacterial culture significantly at two different time points (the fist and third time points). Compared with the past researches, the present study demonstrated that age might be an independent factor of PPM implantation infection. Furthermore, aging resulted in the significant increasing of infection rate after implantation surgery.

### 3.2. The Construction of Positive Strains in the Different Time Points

In the present study, we tried to investigate which strain is the major source of implantation infection. With the bacterial culture test, we analysed the construction of positive strains in the three different time points. The results were showed in Table 3; compared with the other strain, the most positive culture results were *Staphylococcus epidermidis* (84% in the first, 66.6% in the second, and 50% in the third time point). With the data, the major source of PPM infection might be *Staphylococcus epidermidis*.

Interestingly, within 25 *Staphylococcus epidermidis* positive patients, 4 patients were the adult and 21 patients were the elderly (*P* < 0.0001). Therefore, the old patients might be easier to be infected by *Staphylococcus epidermidis*.

### 3.3. Aging Alleviated the Protective Effects of Rinse Significantly

Both previous and present studies indicated that rinse might alleviate positive ratio of bacterial culture significantly. Surprisingly, in the present trial, we further found that the protective effects of rinse are significant, but no significant differences were found in the elderly between prior to and after rinse. In contrast, there was a significant difference in the adult patients between prior to and after rinse, and rinse alleviated positive ratio of bacterial culture significantly. The results were showed in Table 4. The result further confirms that age might be an independent risk factor to PPM-induced infection. Our further analysis of elderly patients found that the presence of diabetes did not affect the results after rinse. The results were showed in Table 5.

## 4. Discussion

PPMs are increasingly being used for the prevention and treatment of various cardiac rhythm disturbances. There were 13 million functioning PPMs worldwide in 2000, and the number of PPM placements has continued to increase. In the United States, there was a 42% increase (from 3.26 per 1000 to 4.64 per 1000) in the cardiac device implantation rate among medicare beneficiaries from 1990 to 1999. In China, to date, there is not an exact number to reflect the PPMs implantation rate [11, 12]. However, it is undoubted that PPMs implantation rate is growing greatly. One of the most feared complications of device placement is infection, which can be associated with substantial morbidity and mortality. Infection rates for these devices reportedly vary from 0.7% to 7.0% [8, 9, 11–13] with a resultant 3.1-fold increase in the number of associated hospitalizations in recent years [13, 14]. Mortality rates attributable to infection have ranged from 2.6% to 3.3% [13–16]. Although recent reports have identified several clinical characteristics associated with developing cardiac device-related infection (CDI), there are limited data on outcomes after treatment. Furthermore, most PPMs were implanted in the elderly. What are the clinical characteristics of PPMs infection in the elderly? It still remained unclear. Several novel observations have been made in the present study. Firstly, we found that there might be a significant positive relationship between age and incidence of PPMs-related infection with a clinical trial. More interestingly, the data indicated that rinse decreased the positive ratio of tissue solution culture; however, the effects only occur in the adult. In the elderly, the protective effects of rinse were alleviated significantly. To our knowledge, no similar results had been published.

The factor associated with the greatest increase in infectious risk in previous study was the occurrence of early noninfectious complication (haematoma, pacemaker dysfunction, and displacement of the intracavity lead), requiring reintervention. Klug et al. reported similar results in the PEOPLE study, and repeated procedures were also shown to increase risk of infection in the Danish registry as well as in a recent study of risk factors for device infection. However, to date, seldom researches are involved in the relationship between age and PPMs infection. Some documents indicated that there was not a significant relationship between age and postimplantation infection. Additionally, the research from Johansen et al. demonstrated an inverse relationship between increasing age and the risk of infection, with the rate of infection being the highest in children and adolescents and declining with age. In present study, the data indicated that age resulted in the significant increasing of tissue culture infection rate. The exact reason that resulted in the above difference is kept unknown. But we found that the present study did not include children and adolescents. In addition, because of the higher cost of ICD and CRT, the implanting number in China is less than that in America/Europe. Therefore, the patients that received ICD/CRT treatments were excluded from the present study. Our finding indicated that age might be an independent risk factor on PMMs implantation infection.

Recently, lots of studies focused on the prevention of PPMs infection. For example, antibiotic is used prior to surgery, which resulted in the cost increasing. The previous works from our lab showed that simple rinse could decrease the infection rate significantly. Furthermore, no significant difference was found between antibiotic and saline rinse. Therefore, 30 mL saline quick rinse will exert an obvious protective effect on implantation-induced infection, which will not increase the cost of treatment. More interestingly, in the present study, we found that age exert is an negative effect on rinse treatment. Aging alleviated the protective effects of rinse.

Rapid growth of the world's geriatric population has heightened awareness of age-related cardiovascular diseases. The elderly are the major population of PPMs implantation. Infection is the most important complication of PPMs implantation. The present study demonstrated that there was the positive relationship between age and PPMs infection. Additionally, we found that saline rinse fail is to alleviate the occurrence of PPM-related infection. The study might be helpful to decrease postimplantation infection in clinical work.

## Figures and Tables

**Table 1:** Baseline characteristics of the study population (mean ± SEM).

	Adult group (*n* = 131)	Elderly group (*n* = 262)	*χ* ^2^	*P*
Age (year)	54.3 ± 10.6	73.2 ± 5.24		
Sex, M/F	72/67	137/125	0.046	0.830
Patients residence (Beijing/other places)	111/20	214/48	0.569	0.451
Nonhealth insurance/health insurance	23/108	53/209	0.400	0.527
HBP/total (*n*/total)	44/131	156/262	23.53	<0.0001
Diabetes (*n*/total)	18/131	63/262	5.668	0.017
Coronary artery disease (*n*/total)	22/131	73/262	5.837	0.016
Causes of PPM implantation				
Sick sinus syndrome (SSS)	63 (48.1%)	133 (50.7%)	2.966	0.0850
AVB (III∘/advanced)	34 (26.0%)	33 (12.6 %)	3.866	0.0493
Permanent atrial fibrillation with long RR interval	27 (20.6%)	47 (17.9 %)	0.04981	0.8234
Battery exhausted	15 (11.5%)	40 (15.3%)	1.834	0.1757
Pacemaker types				
Single chamber	30 (22.9%)	47 (17.9%)	1.365	0.243
Dual chamber	101 (77.1%)	215 (82.1%)		
Number of implant				
One	111 (84.7%)	206 (78.6%)		0.284*
≥2	20 (15.3%)	56 (21.4 %)		

HBP: high blood pressure; M: male; F: female.

*Exact probability.

**Table 2:** The relationship between five factors and positive ratio of sample bacterial culture.

Factors	The first sampling point			The second sampling point			The third sampling point		
	Positive number (positive ratio)	*χ* ^2^	*P*	Positive number (positive ratio)	*χ* ^2^	*P*	Positive number (positive ratio)	*χ* ^2^	*P*
Gender		1.031	0.310		1.157	0.561		0.852	0.356
Male: 204	21 (10.3)			28 (13.7)			18 (8.8)		
Female: 189	22 (11.6)			23 (12.2)			12 (6.3)		

Age		4.057	0.026*		0.501	0.778		4.060	0.045*
<65: 131	8 (7.6)			17 (13.0)			5 (3.8)		
≥65: 262	35 (12.6)			34 (13.0)			25 (9.3)		

BP		2.509	0.113			0.595		0.010	0.919
HBP: 200	26 (13.0)			25 (12.5)			15 (7.5)		
Normal BP: 193	17 (8.8)			26 (13.5)			15 (7.8)		

Blood glucose		0.690	0.408		12.593	0.002		0.309	0.578
Diabetes: 81	11 (13.6)			20 (24.7)			5 (6.2)		
Nondiabetes: 312	32 (10.3)			31 (9.9)			25 (8.0)		

CAD									
CAD: 95	10 (10.5)	0.473	0.492	12 (12.6)	0.330	0.846	9 (9.5)	0.602	0.438
Non-CAD: 298	33 (11.1)			39 (13.1)			21 (7.7)		

*P values <0.05 were considered statistically significant.

**Table 3:** The construction of positive straines in the different time point.

Bacterial strain	The first sampling point		The second sampling point		The third sampling point	
	Positive number	Positive ratio	Positive number	Positive ratio	Positive number	Positive ratio
*Staphylococcus epidermidis *	36***	83.7	34***	66.7	15**	50.0
*Staphylococcus capitis *	4	9.3	8	15.7	2	6.7
*Staphylococcus haemolyticus *	1	2.3	3	5.9	0	0.0
*Staphylococcus aureus *	0	0	3	5.9	0	0.0
*Staphylococcus hominis *	0	0	2	3.8	6	20.0
*Staphylococcus lentus *	0	0	0	0.0	4	13.3
*Staphylococcus intermedius *	2	4.7	1	2.0	3	10.0

Total	43	100	51	100	30	100

**P < 0.01, ***P < 0.001 versus the other strain.

**Table 4:** Aging alleviated the protective effects of rinse.

	Prior to rinse		After rinse		*χ* ^2^	*P*
	Positive number	Positive ratio	Positive number	Positive ratio		
<65 (*n* = 131)	17	13.0	5	3.8	7.145	0.0075*
≥65 (*n* = 262)	34	13.0	25	9.5	1.547	0.2136

*P < 0.05 versus post-rinse.

**Table 5:** Diabetes does not affect the protective effects of rinse in elder patients.

	Prior to rinse		After rinse		χ^2^	*P*
	Positive number	Positive ratio	Positive number	Positive ratio		
Diabetes (*n* = 63)	9	14.3	7	11.1	0.294	>0.05
Nondiabetes (*n* = 199)	25	12.6	18	9.0	1.283	>0.05
